# Case of Compound Comminuted Fracture

**Published:** 1856-01

**Authors:** A. M. Carpenter


### CASE OF COMPOUND COMMINUTED FRACTURE. KEOKUK CITY HOSPITAL REPORT.

BY A. M. CARPENTER, M. D.

On the 29th of August, James McGrew, aged thirty, of Irish descent, sustained an injury upon the right leg, caused by a fall through the hatch of a four story building from the third flour down to the first. In his passage down he came in contact with a large iron hook appended to a block, for the purpose of drawing up large weights, which took effect in the right leg some three inches below the knee or about its upper third, penetrating and tearing out the whole of the muscular substance in that region as well as the blood vessels, and coming in direct contact, fractured both bones of the leg, two and a half inches below their condyles, making its exit upon the outer aspect of the limb, leaving a most hideous wound, encircling it for more than two-thirds of its circumference; he sustained also a slight wound upon the frontal, and one upon the occipital region—together with a compound fracture of the second phalanx of the middle toe. Drs. Hughes, M'Gugin, Allen, and myself were called, who severally and together probed and examined the wound and fracture, and after counsel, it was agreed to remove him to the City Hospital, whereupon the leg was more critically examined and several spicula of detached bone extracted—the wound was accordingly cleansed and the lips brought in apposition by the continuous suture. The fractured bones were likewise properly adjusted and confined by a compress and roller, and the leg was then placed in the best position, observing the suitable amount of extension and counter-extension. Reaction having been established, the patient was ordered an anodyne, Liq. Morphia gtt. xxx, and left for the night.

30th. Visit mane. Had a fair night's rest; pulse 99 and firm; skin warm; tongue thinly coated; temperature in limb equable; foot same and no tumefaction, no untoward symptoms. Limb examined, aspect of wound healthy, slight hemorrhage, bones in apposition. The wound being so extensive and the fracture, as we conceived, in all possibility, extended to the condyles of the bones and involved the joint, more or less, together with the extreme hot weather, guaranteed the suggestion of amputation, to which the patient remonstrated bitterly, swearing that he would crawl from the house. The idea was abandoned and an attempt entered upon to save it if possible. Treatment, adhesive strips, roller from toes up to the knee, over which was applied a second roller extending to the middle third of the thigh, with a binder's board splint, upon the outer aspect of the limb. The limb was then placed and retained in the ordinary surgical box. Was ordered the Saline and Antimonial Mist as a laxative antiphlogistic and controller of the circulatory powers.

Visit vesper. Pulse 115, skin rather warm, but moist, a little restless, slept a couple of hours in the forenoon, no dejections as yet, some pain in the limb. Was ordered the continuance of the Saline Mist, and if not effecting an evacuation in a few hours, to give an enema of Chlorid Sodium and warm water.

Aug. 31st. Visit mane. Aspect better, skin comfortable, tongue improved, one dejection, pulse 102, slept moderately, manifested some mental aberration, appetite good, no unusual swelling of limb. Water dressing and general treatment continued.

Visit vesper. Surface warm, pulse 115, temperature in limb equable, some pain, no dejection since morning, ordered Ol Ricini one ounce and a half, if not efficient, to be followed by an enema. Anodyne, Pul. Dover, grs. x at night, water dressing to be continued.

Sept. 1st. Visit mane. Had a comfortable night, aspect improved, spirits elevated, pulse 102, tongue cleaning, skin warm and pliable, temperature of limb normal, no excess of tumefaction, bowels open, appetite good, wound examined, aspect healthy, and granulating at edges, no suppuration, dressing reapplied and general treatment continued. Toe examined, point of the fractured extremity protruding, which justified a removal by excision through the joint. Treatment, pledgit of lint, roller and water dressing.

Visit vesper. Rested well through the day, no consequent pain, pulse normal and 112, tongue presents no changes, leg doing well, no unnatural heat or Oedema, treatment continued.

Sept. 2d. Visit mane. Pulse normal and 100, tongue looks well, had two copious dejections, skin warm, appetite good, brain clear, patient expresses himself as getting well fast, no remarks upon the limb, treatment continued.

Visit vesper. Nothing unfavorable, no changes in treatment.

Sept. 3d. Visit mane. Aspect not so good, slept about half the night under an anodyne, pulse reduced in frequency and force to 86, however not assignable to want of action in the system, but to action of the antimony, temperature increased, some augmentation of swelling, one dejection copious, tongue looks well, brain not exactly clear, bandage removed, pitting upon pressure in calf and ankle, aspect of wound, at one point suppurating slightly, at another granulating, at another are erysipelatous puff, dressing renewed and general measures continued.

Visit vesper. Symptoms somewhat ameliorated, more tranquil than at morning, spirits more animated, tongue presents no changes, pulse 100 and soft, one dejection, treatment continued.

Sept. 4th. Visit mane. Aspect improved, passed a good night under an anodyne, mind clear, quite buoyant in spirits, occasionally venting Irish sagacity, tongue fair, pulse 90, skin mellow, bowels cleared, no metorism, appetite good, takes tea and dry bread for breakfast, is allowed chicken soup for dinner, for supper relishes tea with roasted apple, temperature in limb and foot of natural standard, complains of comparatively little pain, pressure of roller equable, gives no pain and is allowed to remain, no dejections since previous evening, was ordered an ounce Ol. Ricini, no other changes in treatment.

Visit vesper. Feels pretty well, no unusual heat of surface, tongue a little clouded, pulse 100 and firm, one stool copious, appetite increasing, was ordered an anodyne through the night, Sal. Mist and water dressing continued.

Sept. 5th. Visit mane. Passed a tolerable night, rather somnolent, tongue, no remarks, pulse 81 and soft, appetite good, brain quiet, limb gives scarcely any pain, no excess of swelling, temperature about natural, two evacuations per night, very copious, no tetor, ordered an anodyne, Sal. Mist to be suspended, and water dressing continued.

Visit vesper. Feels very comfortable, slept several hours, pulse a little excited and 92, complains of hunger, bowels quiet, tongue as usual, some odor from leg which was undressed, wound suppurating finely, no increase of Oedema above or below the knee, swelling in the calf rather diminished, roller renewed, resumed the Saline Mist, water dressing continued, with an anodyne.

Sept. 6th. Visit mane. Aspect uneasy, rested badly, pulse 88 and full, tongue clear, one dejection copious, brain clear, complains of good deal of pain in limb, due to its altered position, no increased heat or tumefaction, position changed, reports feeling better, some chilliness owing to approaching suppuration, treatment continued.

Visit vesper. No particular change noted except in the pulse, which is always increased in frequency at the decline of the day, no unusual heat of surface, feels quite comfortable, no alteration in prescription.

Sept. 7th. Visit mane. Aspect good, reports having had an unpleasant night, but the inmates report him as having slept well, tongue clean, pulse 98, surface warm and moist, brain free, some fetor from limb, dressing removed, wound healthy and suppurating throughout its entire extent, pain slight, no augmentation of Oedema in knee or calf, infiltration at ankle increasing, dressing renewed and treatment continued.

Visit vesper. Some increase of soreness, pulse 109, considerable heat about the knee joint, temperature of ankle normal, no ecchymosis at any point, bowels open, ordered an opiate through the night, and the Mist to be suspended.

Sept. 8th. Visit mane. Feels quite comfortable, had a good night, brain composed, muscular strength but little impaired, sits up for several hours during the day, pulse 110 and full, tongue looks well, one copious dejection, skin pleasant, temperature in limb equalized, nothing untoward and no change in treatment.

Visit vesper. Not so well, complains of pain, very restless, aspect uneasy, some excitement, pulse feeble and increased in frequency to 120, tongue colored, bowels lax, skin hot, head clear, appetite good, limb examined, temperature proper, no excess of heat about the joint, slight gaseous crepitation in the thigh, and easily displaced, some evidences of supervening irritative fever, was ordered Calomel and Opii. with a view of arousing the liver, and allaying the irritability then threatened.

Sept. 9th. Visit mane. Reports better, aspect improved, passed quite a good night, one dejection, skin moist, no febrile action, pulse reduced to 100, though feeble, wound suppurating freely, some sloughing of its lips and jagged edges, granulations quite abundant, treatment continued.

Visit vesper. Feels somewhat exhausted from the extreme heat of the day, pulse 113 and compressible, skin comfortably warm, febrile movement smartly abated, some pain in the region of the wound, consequent upon sloughing of the edges, thereby exposing the bellies of the gastrocnemiis to the pressure of the roller, treatment Calomel and Opii., to be repeated as it had failed to excite sufficient action upon the liver, and had not tranquilized the nervous system or induced that amount of rest which the patient greatly demanded.

Sept. 10th. Visit mane. Reports feeling quite comfortable, slept moderately, skin moist, and of pleasant temperature, pulse 105 and compressible, medicine acted happily, bowels soft and well emptied, no excitement of system, maintains strength admirably, is able to sit up and regulate the syphon, or water his limb by means of a dipper adapted for the purpose, foot and leg of natural warmth, was ordered nothing but good diet and cold ablutions to limb.

Visit vesper. Had a pleasant day, but little pain or uneasiness, tongue looks well, had two evacuations, pulse 120, was ordered Liq. Morph. through the night, as he has great difficulty in composing himself.

Sept. 11th. Visit mane. Aspect good, reports feeling as fine as possible, rested well during the night, brain clear, pulse reduced to 99, appetite pretty good, one dejection, roller removed, wound emitting pus freely and quite laudable, smells loud, surface of wound casting healthy granulations, sinuses being formed at the superior and inner aspect of the wound, remainder closing up, marked tenderness in knee joint, treatment continued.

Visit vesper. Not feeling so well, slept some through the day, tenderness in joint very acute, pulse 118 and small, some febrile action, bowels a little full, was ordered Hydrg. Chlor. Mist, grs. v, Pul. Opii. gr. i per night, general measures continued.

Sept. 12th. Visit mane. Expression better, reposed quietly during the night, two evacuations, pulse reduced to 109, and increased in volume, skin pleasantly warm, was prescribed nothing internally, ablutions continued.

Visit vesper. Complaining smartly, some febrile movement, pulse 120 and small, was prescribed an anodyne, general treatment continued.

Sept. 13th. Visit mane. Reports feeling pretty badly, rested poorly through the night, pulse 110 and softer than usual, tongue presents nothing of note, three dejections, skin moist, wound suppurating largely, granulations becoming fungus.

Visit vesper. No material change in the general symptoms, knee quite sensitive upon pressure, slight shivering, indicative of suppuration, pulse 119, was ordered an anodyne, water dressing continued.

Sept. 14th. Visit mane. Aspect good, had a tolerable night, pulse 105 and regular, skin warm, temperature natural, two dejections copious, appetite fair, complains of pain in the back and shoulders, right arm paralyzed from the constrained recumbent posture, treatment continued.

Visit vesper. Expresses himself as feeling very poorly, aspect anxious, skin hot, pulse 120 and small, irritative fever unavoidable, bowels quick, prescribed an anodyne through the night.

Sept. 15th. Visit mane. Aspect uneasy, very restless, spent a disagreeable night, pain in the knee increasing, considerable fever, pus evidently absorbed into the economy, pulse 115 and thready, one dejection, temperature in limb normal, wound looked to, aspect flattering, suppuration extensive, pus exhibiting some tendency to sacculate, upon pressure in direction of the sinuses, some three ounces of pus was evacuated, was ordered Cal. and Opii., full dose, general treatment continued.

Visit vesper. Condition pretty much the same, with the exception of pulse a little excited and 120, skin hot, was ordered an anodyne.

Sept. 16th. Visit mane. Expresses himself as having had a terrible night, aspect uneasy, tongue clean, skin hot, pulse 125 and imparting a thrill to the feel, physical power a little impaired, three dejections, rather thin, temperature in limb not augmented, great thirst, brain clear, was prescribed a full anodyne, water dressing continued.

Visit vesper. More composed, reposed some under an Opiate, pulse 120 and evinces some excitement of system, aspect of wound healthy, discharging copiously, tendency to an issue in the posterior and middle aspect of the limb, pressure was made upon the point and the matter thereby forced up in the direction of the sinus, treatment, a many tail bandage was applied, with compresses over the depending point to prevent the matter from migrating down the limb and interfering with the healthy tissues, was ordered an astringent with anodyne, water dressing continued.

Sept. 17th. Visit mane. Had an unpleasant night, brain excited, skin a little warm, pulse reduced to 112 and more yielding, bowels loose, three dejections, limb tolerably comfortable, was put upon Hydrg. Cum. Creta, Quinia, and Doveri, ablutions continued.

Visit vesper. Symptoms much improved, rested well during the day, bowels constrained, skin warm and moist, but little fever, pulse 118, treatment continued.

Sept. 18th. Visit mane. Reports feeling better, aspect improved, slept admirably, brain quiet, no febrile action, pulse 115, temperature regular, wound examined, granulations protruding, limb somewhat enlarged, pressure was used and one pint of pus discharged, many tail bandage failing to steady and support the limb, was dispensed, and the adhesive strips and roller resumed, treatment continued.

Visit vesper. Feels quite weak, general symptoms as usual, pulse 120, considerable pain in limb, bowels rather lax, dejections serous, some irritability of mucus lining, skin quite warm, appetite impaired, diet, essence of beef, was ordered a little wine, usual measures continued.

Sept. 19th. Visit mane. Symptoms improved, pulse 110 and regular, skin comfortably warm, no fever, wound examined, granulations depressed to the level of the surface under the influence of strips, quantity of pus discharged, some sensitiveness in the joint and extending up the thigh as far as the superior spinous process of the Ilium, roller was reapplied, leaving the wound and sinus free, treatment continued.

Visit vesper. Condition much the same, pulse 120, bowels quiet, slight fever, no additional prescription.

Sept. 20th. Visit mane. Aspect dejected, had a restless night, pain increasing above the knee, pulse 111 and feeble, two dejections, skin moist and cool, sacrum abraded, was ordered Doveri and Quinia, wine and water dressing continued.

Visit vesper. Feels moderately comfortable, no sensible changes since morning, treatment continued.

Sept. 21st. Visit mane. Reports better, spent a good night, pulse 109, one dejection, strength improved, appetite improving a little, urine passes freely, ordered nothing additional.

Visit vesper. No particular changes, skin warmer and circulation more active, as usual in the evening.

Sept. 22d. Visit mane. Expression not so good, pulse 125 and small, three dejections copious and watery, much prostrated, limb examined, swelling increasing above the knee, matter forming in the popliteal space, fluctuation very distinct, pressure being exerted, the sac burst and the matter found its exit through the joint to the sinus, amounting to near a pint, outer aspect of thigh red, congested and sensitive, extensive formation of pus under Fascia Lata of thigh, treatment, roller applied from groin down to knee, ordered Pul. Opii. gr. i, Tannic Acid gr. i, Pul. Camphor grs. ii, every third hour or according to circumstances, with wine freely.

Visit vesper. General condition about the same, skin rather moist, pulse 120, slept an hour or two during the day, no alteration in prescription.

Sept. 23d. Visit mane. Reports feeling badly, dozed a little through the night, aspect sunken and wishful, skin warm, pulse 119, three dejections, excessive pain in knee joint, thigh, outer aspect inflamed and sensitive, maintains strength well, is still able to sit up, appetite impaired, was prescribed wine whey and soup as diet, port wine and astringents continued.

Visit vesper. Limb quite painful, thigh much distended, enormous discharge of pus during the day, upon elevating the limb the matter made its way per the joint and out the sinus in a forced stream, wound looks well, nourishing and stimulating measures continued.

Sept. 24th. Visit mane. Feels weak, passed a tolerable night, dozed now and then, aspect vacant, spirits depressed, skin moist, and not unfrequently beaded with perspiration, tongue dry, pulse 122 and feeble, nourishment becoming loathsome, two dejections, leg painful, thigh much distended, pus evidently seeking exit, a bistoury was plunged into the inferior portion of the limb through the Fascia Lata, thereby giving vent to a large quantity of pus, amounting to half a gallon and three gills, relieving the distension and consequent suffering, roller reapplied, ordinary measures continued.

Visit vesper. Symptoms bettered, pulse diminished in frequency to 119, febrile movement abating, skin moist and pleasant, slept some, bowels quiet, limb moderately comfortable, no diminution in strength, pus welling out of the fresh incision, prescribed Cod Liver oil with a view to its nutrient effect, in doses of half teaspoonful thrice in the 24 hours and increased if tolerated by the stomach, local and general treatment continued.

Sept. 25th. Visit mane. Looks much better, feels, as expressed, pretty sharp, had a good night, skin perspiring, pulse 122, bowels soluble, one stool, brain free, appetite returning, limb less painful and diminished greatly, treatment, local no change, general continued, with the addition of barks, Cod oil discontinued.

Visit vesper. Feels pretty comfortable, but little pain, discharging freely, skin pliable, pulse 120, bowels quiet, ordered anodyne at night as usual.

Sept. 26th. Visit mane. Reports feeling as fine as silk, spirits aroused, strength improved, appetite augmenting, is able to sit up, still remonstrates against the idea of removal of his limb as at first, surface pleasantly warm, pulse reduced in frequency to 110 and more compressible, tongue less dry, thirst diminishes as does the drainage, which is yet considerable, from both openings, dressings not disturbed, local and general treatment continued.

Visit vesper. Patient feels first rate, and entertains the fairest hopes of his recovery, no other remarks, treatment continued.

Sept. 27th. Visit mane. Had a passable night, feels tolerably well, strength and appetite still augmenting, had slightly ulcerated although well padded, surface warm, pulse 112, three dejections, wound looks healthy, as well as contiguous surface, discharge most profuse, though patient's system counteracts it amazingly, treatment, barks affecting the bowels discontinued and put upon Chlorine mixture, general course continued.

Visit vesper. No material changes, pulse 114, surface agreeably warm, treatment continued.

Sept. 28th. Visit mane. Reports having passed a tolerable night, slept occasionally, skin pleasant, brain clear, pulse 106 and feeble, complains of great pain throughout the limb, discharging largely, treatment continued.

Visit vesper. Some febrile action, pulse 120 and excited, slept well during the day, one dejection, treatment continued.

Sept. 29th. Visit mane. Had a bad night, brain excited, pulse 107, skin free from heat, two dejections, appetite scant, discharging profuse, wound healthy, knee much enlarged, dressings renewed and treatment continued.

Visit vesper. Feels middling well, pulse 120, skin gently perspiring, one dejection, no change.

Sept. 30th. Visit mane. Feels badly, dozed some per night, pulse 113, surface warm, sinuses discharging freely, crepitation in joint, was ordered an anodyne, treatment continued.

Visit vesper. Nothing unusual to remark, appetite moderate, takes beef essence, tea, and dry bread.

Oct. 1st. Visit mane. Aspect thin, had a tolerably comfortable night, manifested slight aberration of mind, surface moist, pulse 103, redness of tongue diminishing, feels very weak, bowels acted twice, tenderness in course of Femur up to groin, pus mixed with a sanguineous fluid, was ordered good diet and wine freely.

Visit vesper. Feels very well, with the exception of weakness, no other remarks.

Oct. 2d. Visit mane. System prostrated, no fever, pulse 112, three dejections, was ordered an Opiate, no other changes.

Visit vesper. Symptoms much the same, stimulant and nourishment continued.

Oct. 3d. Visit mane. Feels very weak, raved through the night, aspect troubled, tongue ex-sanguined, pulse 125 and fluttering, extremities cool, bowels lax, limb examined, evidences of secondary hemorrhage, coagula oozing from the sinus, several spicula of bone extracted, treatment involved nothing but sustaining measures.

Visit vesper. Dissolution speedily threatened, pulse rapidly failing, dejections involuntary, drinks quart of wine in the 24 hours.

Oct. 4th. Visit mane. Aspect rather frightful, hemorrhage persistent, great thirst for wine.

Visit vesper. Patient in a moribund state, treatment, assist him to die as comfortably as possible.

5th. An autopsy revealed extensive fracture of the Tibia and Fibula, with a vertical crack of the inner condyle of the Femur, extending to the middle of the shaft.

